# A Professor in the Chair

**Published:** 1861-07

**Authors:** 


A PROFESSOR IN THE CHAIR.—"The nobility of science, as contrasted with the nobility of birth," says the *London Lancet* of May fourth, "was honorably and successfully recognized this week by our newly-adopted professional brethren, the dental surgeons. The first anniversary dinner of the Dental Hospital was presided over, not by a duke, nor an earl, nor a marquis—not by a cabinet minister nor a political celebrity. The chair was taken by Professor Owen; and in his person were aptly represented the highest claims which odontological science can put forth. The attendance was more numerous than has been seen at an hospital dinner for many a day. Not only was the dignity of science vindicated by the selection of the president for the first anniversary meeting, but the perfect fitness and adaptability of a man of the highest scientific attainments for this kind of social responsibility were very triumphantly demonstrated. The number of guests attracted far exceeded anticipations, and the amount of subscriptions overpassed them in a twofold measure. We hope that this incident may afford a precedent, and that at similar meetings distinguished members of the profession may be called to preside with like felicity and equal success."

